# Effects of acupuncture and moxibustion therapy on endometrial receptivity of infertile women with *in vitro* fertilization–embryo transfer: status quo and countermeasures

**DOI:** 10.3389/fmed.2025.1702214

**Published:** 2026-02-10

**Authors:** Xiao-Zhu Guo, Zhen Qin, Deng-Hui Wu, Han Han, Yan-Hua Han, Xiao-Ke Wu, Yue-Hui Zhang

**Affiliations:** 1 Heilongjiang University of Chinese Medicine, Harbin, China; 2 Department of Obstetrics and Gynecology, First Affiliated Hospital, Heilongjiang University of Chinese Medicine, Harbin, China; 3 Wuxing District People's Hospital, Huzhou, Zhejiang, China; 4 First Affiliated Hospital, Heilongjiang University of Chinese Medicine, Harbin, Heilongjiang, China; 5 The First Affiliated Hospital of Harbin University, Harbin, China

**Keywords:** acupuncture and moxibustion, biomarkers, endometrial receptivity, infertility, IVF-ET

## Abstract

**Background:**

*In vitro* fertilization–embryo transfer (IVF-ET) is a core assisted reproductive technology (ART) for infertile couples, but poor endometrial receptivity (ER) severely impairs embryo implantation and reduces success rates, imposing significant psychological and economic burdens. Acupuncture and moxibustion have been validated to improve ER via multiple mechanisms, yet no reviews systematically summarize clinical evidence on their subtypes and underlying pathways to guide future research.

**Objective:**

This study aims to comprehensively synthesize recent advances in acupuncture and moxibustion for enhancing ER in IVF-ET patients, promoting clinical application and research awareness.

**Methods:**

A systematic literature search was conducted across PubMed, Web of Science, CNKI, and WanFang databases (January 2020–June 2025), supplemented with seminal studies. After screening, 214 studies were included for qualitative synthesis; methodological quality was assessed using RoB 2.0 (RCTs) and NOS (non-RCTs).

**Results:**

Acupuncture (traditional manual, electroacupuncture, and TEAS) and moxibustion (wheat, herbal cake, and warm uterus moxibustion) improve ER by regulating endometrial thickness, blood flow (e.g., reduced PI/RI), hormone levels (E2 and P), and type A endometrium ratio. Combined therapies (e.g., warm needling + acupuncture) further enhance efficacy. Mechanisms involve modulating autophagy (AMPK/mTOR pathway), pinopode-related molecules (LIF, integrin αvβ3), non-coding RNAs (miR-449a, circRNAs), and VEGF-mediated angiogenesis.

**Conclusion:**

Acupuncture and moxibustion are promising adjunctive therapies for ER enhancement in IVF-ET, but high-quality, standardized RCTs are needed to confirm efficacy, standardize protocols, and reduce bias.

## 1 Introduction

Infertility refers to a functionally impaired disease that affects up to 15% of childbearing-age couples globally (1, 2). In China, the incidence of infertility is 25%, which is higher than the global standard (3). Additionally, approximately 5% of couples are ultimately unable to conceive naturally (4). The causes of infertility are complex and equally occupied by male and female factors in general (5), among which, ovulatory dysfunction (represented by polycystic ovary syndrome, PCOS), endometriosis (EMs), and fallopian tube diseases (such as hydrosalpinx) are considered the most common female factors attributed to infertility, accounting for 25%, 15%, and 11%, respectively (6–8). IVF-ET is a common treatment option for women with fertility needs, in cases where natural conception is not possible (9, 10). Compared to other ART methods, such as unstimulated intrauterine insemination (IUI) with gonadotropins, IVF-ET has a higher live birth rate (LBR), though considered invasive, risky, and expensive (11). Relevant studies have shown that the common diseases stated previously and unexplained infertility can contribute to impaired ER (12–15), which affects the process of ET (16), and ultimately reduces the success rate of IVF-ET.

ER is the ability of the endometrium to facilitate normal embryo implantation, allowing the embryo to attach, invade, and develop into a new individual (17). Normal ER requires adequate open time for the window of implantation (WOI), typically 4 days, which occurs 7–10 days post-ovulation (18). Embryo implantation can only occur during this short period of each cycle due to dynamic human endometrial changes (19). Recurrent implantation failure (RIF) is a common clinical phenomenon in IVF-ET, defined as the failure to achieve a clinical pregnancy in women under 40 years after the transfer of at least four high-quality embryos in at least three fresh or frozen cycles (20), affecting approximately 15% of couples (21). Poor ER-induced asynchronous/pathological windows of implantation have already been confirmed as one of the triggers of RIF (22). Women with RIF are usually anxious and depressed (23, 24), and repeated infertility treatments may hinder women's career development (25), lower their social status, increase psychological pressure, and ultimately reduce their quality of life (QoL). Research has shown that mental stress during early pregnancy affects the functional status of ER by impairing the adrenergic receptor signaling (26), which directly harms female fertility (27). Accordingly, the success rate of IVF-ET may be increased by enhancing the ER, thereby improving pregnancy outcomes, and reducing or even avoiding RIF (28).

**Figure 1:**
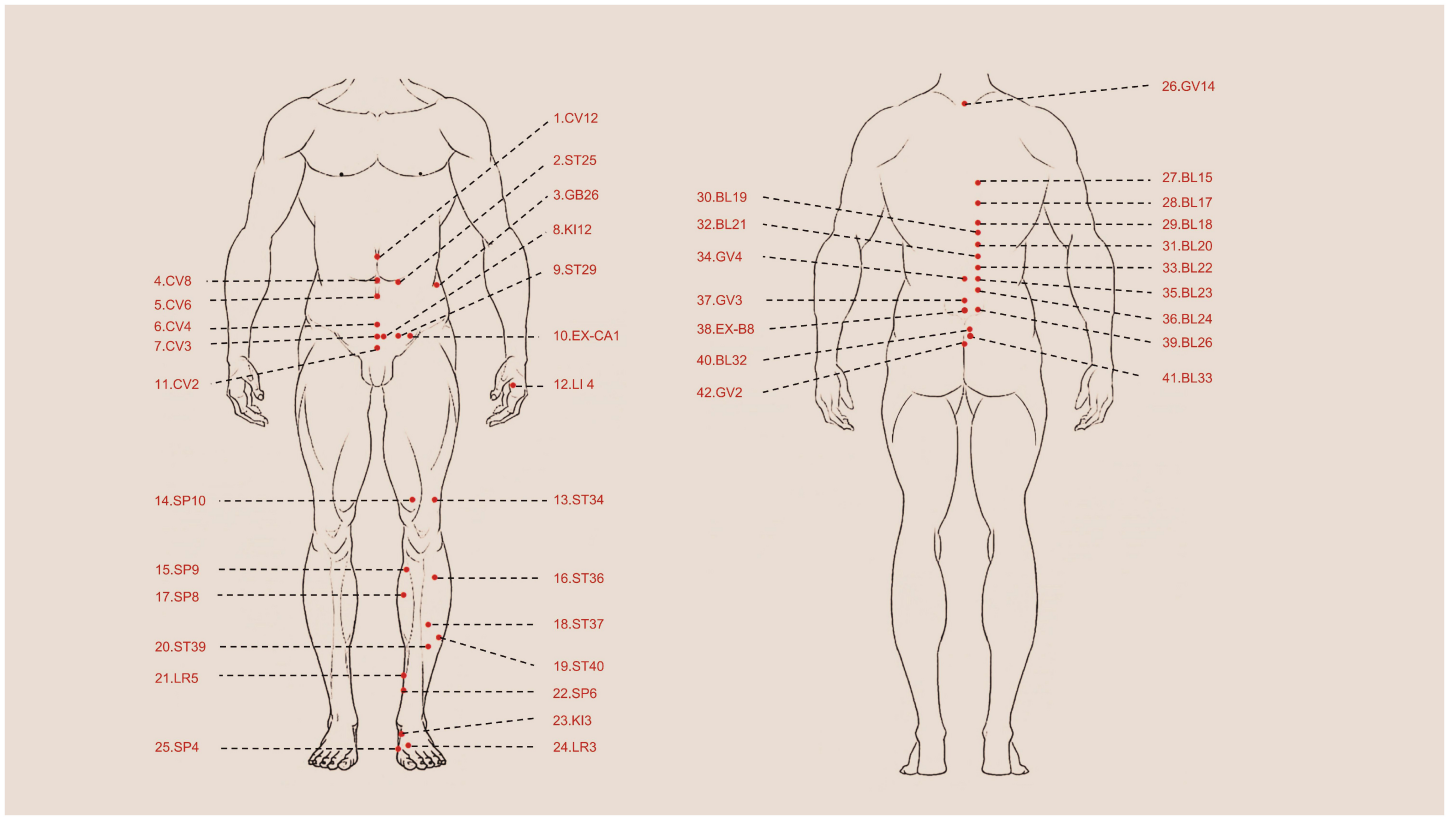
Summary of acupoints in clinical research. 1. CV12 Zhongwan, 2. ST25 Tianshu, 3. GB26 Daimai, 4. CV8 Shenque, 5. CV6 Qihai, 6. CV4 Guanyuan, 7. CV3 Zhongji, 8. KI12 Dahe, 9. ST29 Guilai, 10. EX-CA1 Zigong, 11. CV2 Qugu, 12. LI4 Hegu, 13. ST34 Liangqiu, 14. SP10 Xuehai, 15. SP9 Yinlingquan, 16. ST36 Zusanli, 17. SP8 Diji, 18. ST37 Shangjuxu, 19. ST40 Fenglong, 20. ST39 Xiajuxu, 21. LR5 Ligou, 22. SP6 Sanyinjiao, 23. KI3 Taixi, 24. LR3 Taichong, 25. SP4 Gongsu, 26. GV14 Dazhui, 27. BL15 Xinshu, 28. BL17 Geshu, 29. BL18 Ganshu, 30. BL19 Danshu, 31. BL20 Pishu, 32. BL21 Weishu, 33. BL22 Sanjiaoshu, 34. GV4 Mingmen, 35. BL23 Shenshu, 36.BL24 Qihaishu, 37. GV3 Yaoyangguan, 38. EX-B8 Shiqizhui, 39. Bl26 Guanyuanshu, 40. BL32 Ciliao, 41. BL33 Zhongliao, 42. GV2 Yaoshu.

Human chorionic gonadotropin (HCG) and the gonadotropin-releasing hormone (GnRH) are commonly used as first-line ovulation stimulants during IVF-ET to promote egg maturation and induce ovulation. However, a high recommended dosage of HCG may result in increased economic burden and the possibility of ovarian hyperstimulation syndrome (OHSS), a severe complication associated with increased capillary permeability (29–31). The GnRH comprises two types: the GnRH agonist (GnRH-a) and the GnRH antagonist (GnRH-A). Studies have shown that GnRH-a can improve ER (32) and decrease the incidence of OHSS when compared with HCG (33). However, the commonly used GnRHa, represented by triptorelin, is expensive (29) and has the potential to trigger OHSS (34). Furthermore, recent research suggests that the frozen embryo transfer (FET) strategy, currently used in clinical practice for infertile women undergoing IVF-ET (35), has clear clinical advantages, including increased cost-effectiveness, optimized ER, and reduced incidence of OHSS and risk of preterm delivery (35–38). Nevertheless, relevant studies have reported its association with some serious diseases, such as hypertensive disorders of pregnancy, and can even develop into pre-eclampsia. To further evaluate its safety, more high-quality randomized controlled trials (RCTs) are needed (35, 38), and it is also necessary to find an adjuvant therapy with precise treatment function, low side effects, and low cost to improve ER and increase CPR (38). Acupuncture and moxibustion therapy are commonly used in clinical practice to treat or prevent diseases by regulating qi and blood to harmonize yin-yang, based on the theories of zang-fu organs, channels, and collaterals, and qi and blood (39). The two aspects of the therapy differ from each other in terms of their operating method and therapeutic mechanisms. Acupuncture therapy aims to regulate and harmonize qi and blood by inserting filiform needles into acupoints to achieve “De qi” and involves dredging the channels and quickening the collaterals through lifting, thrusting, twirling, and needle retention methods. On the other hand, moxibustion therapy applies warm stimulation to acupoints or uncomfortable parts of the body by igniting moxa cones or other combustible materials to warm and unblock the channels and collaterals. The use of acupuncture and moxibustion therapy in ART can be traced back to a study published in 2002 (40), and since then, a growing number of studies have been conducted annually. Traditional Chinese Medicine (TCM) holds the belief that poor ER is attributed to kidney deficiency (41). According to TCM theory, the lumbus is the house of the kidney; accordingly, the selected acupoints mainly focus on the foot shaoyin kidney channel, back, and waist (Figure 1). Relevant studies have demonstrated that acupuncture and moxibustion therapy can improve ER-related indices in a dose-dependent manner with limited side effects (42, 43) and even serve as an independent intervention for infertile women not receiving ART (44). Other studies have also reported that acupuncture and moxibustion therapy can reduce anxiety in infertile women during ET, thereby improving their psychological status (45–47). Importantly, it can also be used to prevent and treat serious complications represented by OHSS (48–51). Acupuncture and moxibustion therapy have unique advantages in providing favorable conditions for ET by improving ER, ultimately increasing the CPR of IVF-ET (52). This review summarizes the latest research progress on PubMed, Web of Science, CNKI, and WanFang databases, from the aspects of clinical and basic research by searching the literature associated with the effect of acupuncture and moxibustion therapy on the ER of infertile women receiving IVF-ET, aiming to pave the way for the following research.

## 2 Method

Relevant literature on the effects and mechanisms of acupuncture and moxibustion therapy on endometrial receptivity in infertile women undergoing *in vitro* fertilization–embryo transfer (IVF-ET) was systematically searched in PubMed, Web of Science, CNKI, and WanFang databases. The search primarily covered publications from January 2020 to June 2025 to capture the latest developments in this field. However, seminal or highly relevant studies before 2020 were also included to ensure theoretical rigor. No language restrictions were applied, but gray literature (e.g., unpublished theses and conference abstracts) was excluded due to limited accessibility and data validity. The search strategy combined the following keywords and their equivalents: “acupuncture,” “moxibustion,” “endometrial receptivity,” and “*in vitro* fertilization-embryo transfer.” Boolean operators (“AND”/“OR”) were used to refine the search.

Inclusion criteria were as follows: (1) Study design: Randomized controlled trials (RCTs), non-RCTs, cohort studies, or mechanism-focused basic research; (2) Population: Infertile women undergoing IVF-ET with ER impairment (e.g., thin endometrium and recurrent implantation failure [RIF]); (3) Intervention: Acupuncture (traditional manual acupuncture, electroacupuncture [EA], and transcutaneous electrical acupoint stimulation [TEAS]) or moxibustion (wheat moxibustion, herbal cake moxibustion, and warm needling) alone or in combination; (4) Outcomes: Clinical indicators (ER-related ultrasound parameters, clinical pregnancy rate [CPR], and live birth rate [LBR]) or mechanistic indicators (autophagy markers and molecular signaling pathway molecules); (5) Full-text available.

Exclusion criteria were as follows: (1) Review articles, case reports, or editorials; (2) Studies without ER-related outcomes; (3) Interventions combining acupuncture/moxibustion with unapproved drugs or therapies; (4) Insufficient data for qualitative synthesis.

**Figure 2:**
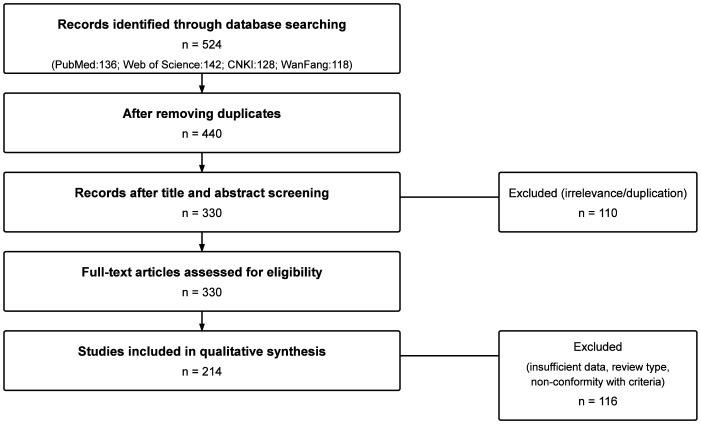
PRISMA flow diagram illustrating the literature identification, screening, eligibility, and inclusion process for studies on acupuncture and moxibustion therapy improving endometrial receptivity in infertile women undergoing IVF-ET.

A total of 524 records were initially identified through database searches, including PubMed (*n* = 136), Web of Science (*n* = 142), CNKI (*n* = 128), and WanFang (*n* = 118). After removing duplicates, 440 records remained. Following title and abstract screening, 110 studies were excluded primarily due to irrelevance. The full texts of 330 articles were then assessed for eligibility, and 116 were excluded for reasons such as insufficient data (*n* = 48), being of review type (*n* = 32), or non-conformity with the predefined inclusion criteria (*n* = 36). Finally, 214 studies were included in the qualitative synthesis (PRISMA flow diagram, Figure 2), ensuring transparency and reproducibility of the selection procedure.

Given that this study is an evidence-based narrative review, the methodological quality of the included trials was qualitatively assessed. Randomized controlled trials (RCTs) were evaluated with reference to the Cochrane Collaboration'ss Risk of Bias Tool (RoB 2.0), while non-randomized studies were examined with reference to the Newcastle–Ottawa Scale (NOS). The risk of bias results are summarized in Supplementary Table S1, with most RCTs showing moderate bias (*n* = 127) due to incomplete blinding, and non-RCTs showing high quality (NOS score ≥7: *n* = 63).

The risk of bias and study design limitations were summarized descriptively to provide an overall assessment of evidence quality and potential methodological weaknesses.

## 3 Clinical research on acupuncture and moxibustion

Current clinical research uses ultrasound diagnostic parameters, primarily related to ER, to confirm the efficacy of acupuncture and moxibustion therapy on infertile women undergoing IVF-ET. These parameters include the thickness, blood flow, volume, and types of endometrium, as well as hormone levels such as estradiol (E2) and progesterone (P) (53, 54). Although endometrial biopsy and endometrial receptivity array (ERA) are available to assess the quality of ER (55, 56), their drawbacks make them unsuitable as regular screening tools, and specific screening tools for ER are still being researched. Endometrial biopsy is considered an invasive procedure with delayed feedback of results and is prohibited during the ET cycle, and ERA is expensive and difficult to evaluate the results (18). Endometrial thickness and the levels of E2 and P are considered to be closely related to CPR during the process of ET (57). Endometrial thickness provides a suitable environment for embryo implantation (58, 59), which is a key indicator of successful implantation and ER evaluation (44). Regarding the levels of E2 and P, they not only regulate the endometrial thickness through periodic changes (60) but also coordinate the molecular and histological changes of the implantation window in a time-dependent manner (61, 62). Embryo implantation success depends on adequate endometrial blood flow, which is supplied by the branch arteries of the uterine spiral artery (63). The resistance index (RI) and pulsatility index (PI) of the uterine spiral artery reflect the endometrial blood perfusion status. It is generally believed that high RI and PI can negatively impact the ET process and, consequently, pregnancy outcomes (63, 64). In addition, the endometrial and sub-endometrial vascularization index (VI), flow index (FI), and vascularization flow index (VFI) can be used to reflect the conditions of endometrial blood flow perfusion and predict the cycle outcomes of IVF-ET (65, 66). Some scholars have emphasized the importance of endometrial volume changes in predicting IVF-ET pregnancy outcomes and ER assessment (67, 68). Successful embryo transfer is predicted when the endometrial volume is within the average range of 2.0–2.5 ml, and when the volume is less than 2.0 ml, there is a decreased chance of clinical pregnancy and embryo implantation rate (EIR) (69). A study found that the endometrial volume, along with sub-endometrial VFI, could replace endometrial thickness as a new parameter combination to predict IVF-ET outcomes (70). Based on the typing standard developed by Gonen et al. (71), the endometrium can be classified into types A, B, and C. Among them, type A endometrium, which has a clear trilaminar pattern, is considered the most suitable for embryo implantation and has the highest CPR (59), making it a useful predictor of pregnancy outcome. The abovementioned factors related to ER are crucial in evaluating ER and predicting IVF-ET pregnancy outcomes. Changes in these factors, which can be modulated by acupuncture and moxibustion therapy, can clarify their impact on the ER of infertile women undergoing IVF-ET.

### 3.1 Acupuncture

#### 3.1.1 Traditional manual acupuncture

For infertile women undergoing IVF-ET, manual acupuncture therapy can be an effective adjuvant treatment to improve pregnancy outcomes after pattern differentiation and acupoint selection (72). However, it is important to note that this treatment should only be administered by acupuncturists with professional knowledge and practical experience to ensure safety and effectiveness. The single-blind RCT conducted by Dong et al. (73) divided 70 infertile women undergoing IVF-ET into two groups and administered 4 days of traditional manual acupuncture and sham acupuncture treatment, respectively, from oocyte aspiration to the day of ET. The traditional manual acupuncture group showed significantly improved VI, VFI, and FI compared with the sham acupuncture group, which directly confirmed the efficacy of traditional manual acupuncture in increasing endometrial blood flow parameters to improve ER in infertile women receiving IVF-ET. Traditional manual acupuncture can also regulate other ER-related factors to improve ER. Xu et al. (74) selected 60 patients diagnosed with anovulatory infertility and receiving FET and randomly assigned them to the observation and control groups. The observation group received manual acupuncture treatment based on the conventional ovulation induction (OI) protocol, beginning on the 2nd day of the menstrual cycle, once every other day until 1 day before undergoing ET, with two groups of acupuncture points and retained needles for 30 min after achieving “De qi” and needle manipulations. After the treatment, the researchers observed improved endometrial thickness and endometrial types on the HCG trigger day in the observation group, with ultimately higher EIR and CPR. In the RCT conducted by Wu et al. (75), 83 infertility patients induced by PCOS who were undergoing IVF-ET were recruited and randomly assigned to an observation group (*n* = 40) and a control group (*n* = 43). The observation group received manual acupuncture treatment based on the conventional GnRH-a long protocol, once every other day from the time of OI until the day of ET. Two sets of acupoints were used, and the needles were retained for 30 min after achieving the “De qi” sensation and needle manipulations. After treatment, the observation group showed lower levels of serum E2 and P, a higher proportion of type A endometrium on the day of HCG injection, and higher CPR. In the single-blind RCT conducted by Wang et al. (76), 60 infertile women undergoing FET were randomly assigned to study and control groups. The study group received manual acupuncture treatment based on conventional estradiol valerate (E2V) treatment from the day after the menstrual period until the ovulatory phase, and both groups were treated for three consecutive menstrual cycles. Results showed higher endometrial volume and maturation time, and lower RI and PI in the study group, in addition to the improved endometrial thickness and pregnancy outcomes having been confirmed in the previous two studies.

**Figure 3:**
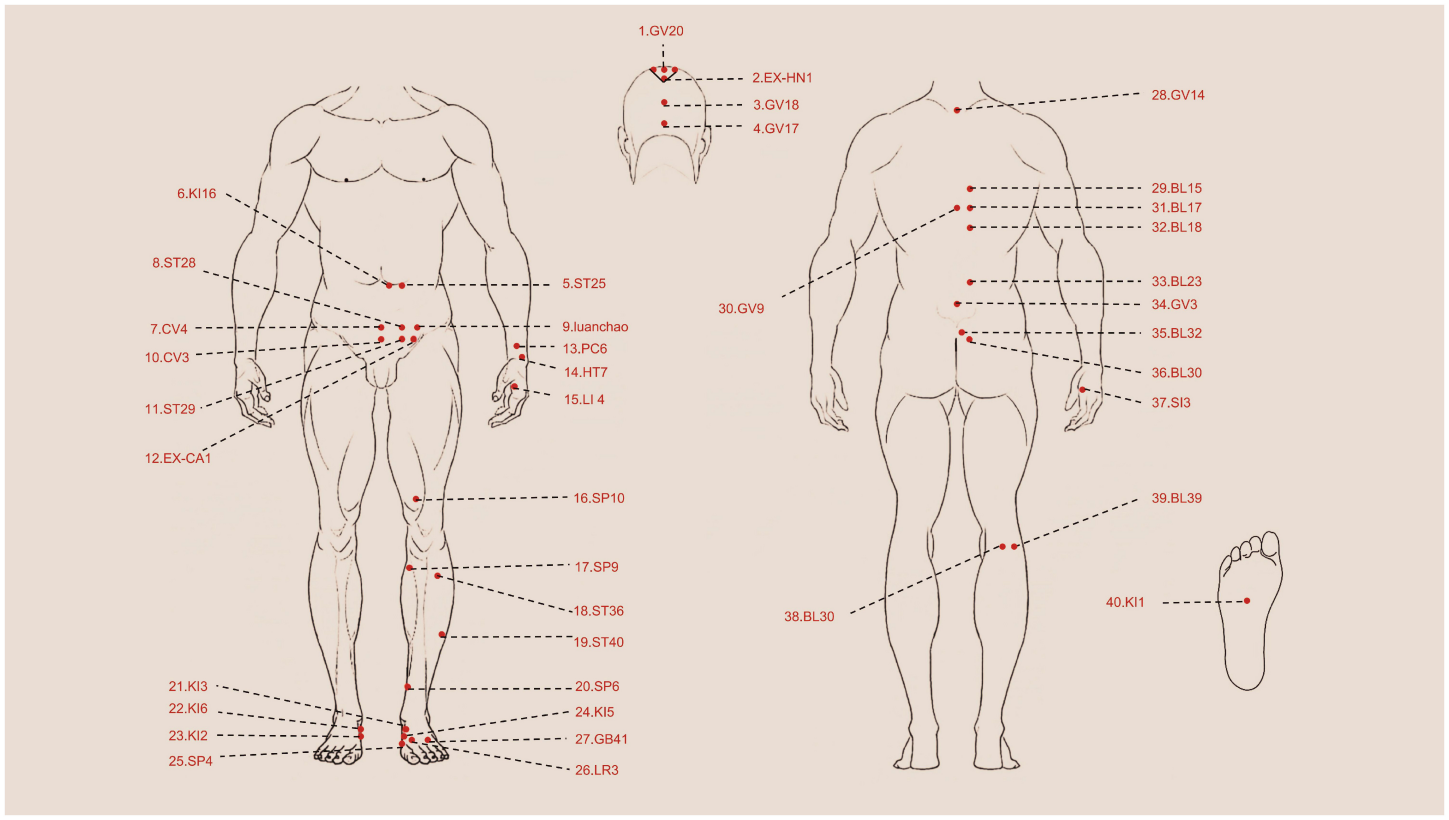
Summary of Acupoints Used in Staged Acupuncture and Moxibustion Therapy. 1. GV20 Baihui, 2. EX-HN1 Sishencong, 3. GV18 Qiangjian, 4. GV17 Naohu, 5. ST25 Tianshu, 6. KI16 Huangshu, 7. CV4 Guanyuan, 8. ST28 Shuidao, 9. Luanchao, 10. CV3 Zhongji, 11. ST29 Guilai, 12. EX-CA1 Zigong, 13. PC6 Neiguan, 14. HT7 Shenmen, 15. LI4 Hegu, 16. SP10 Xuehai, 17. SP9 Yinlingquan, 18. ST36 Zusanli, 19.ST40 Fenglong, 20. SP6 Sanyinjiao, 21. KI3 Taixi, 22. KI6 Zhaohai, 23. KI2 Rangu, 24. KI5 Shuiquan, 25. SP4 Gongsun, 26. LR3 Taichong, 27. GB41 Zulinqi, 28. GV14 Dazhui, 29. BL15 Xinshu, 30. GV9 Zhiyang, 31. BL17 Geshu, 32. BL18 Ganshu, 33. BL23 Shenshu, 34. GV3 Yaoyangguan, 35. BL32 Ciliao, 36. BL30 Baihuanshu, 37. SI3 Houxi, 38. BL40 Weizhong, 39. BL39 Weiyang, 40. KI1 Yongquan.

In summary, these studies indicate that manual acupuncture therapy can improve ER-related parameters, including endometrial thickness, volume, blood flow, and hormonal levels, which may improve IVF-ET pregnancy outcomes based on the Western medical OI/endometrial preparation protocol (Table 1). Recent studies have adopted a TCM theory-based, systematic, and scientific acupuncture and moxibustion therapy protocol for IVF-ET patients, which involves the application of staged acupuncture and/or moxibustion therapy with selected acupoint combinations based on the variation in the waxing and waning of yin and yang in different phases of the menstrual cycle, including the menstrual period, ovulatory phase, follicular phase and luteal phase, to improve the effectiveness of IVF-ET treatments. Xing et al. (77) randomly divided 72 recruited patients into acupuncture and control groups. The acupuncture group received three consecutive menstrual cycles of staged manual acupuncture intervention based on the FET protocol, starting from the second menstrual cycle after recruitment, three times a week. The study found that the acupuncture group had significantly increased endometrial thickness and ratios of type A and B endometrium, and regulated levels of follicle-stimulating hormone (FSH), luteinizing hormone (LH), LH/FSH, testosterone (T), and E2. Importantly, the acupuncture group showed increased CPR and LBR. Compared to traditional manual acupuncture, staged acupuncture comprehensively modulates the key factors related to ER in infertile women receiving IVF-ET, according to the periodic changes in the menstrual cycle, to improve pregnancy outcomes (Figure 3). Its distinctive advantages reflect the continuous development and progress of acupuncture therapy.

**Table 1:** Summary of the clinical research of acupuncture.

**Type of acupuncture**	**Study design/disease**	**ART type**	**Intervention description**	**Treatment course**	**Main acupoints**	**Technique/manipulation**	**Key findings**	**References**
Traditional manual acupuncture	Single-blind RCT/not specified	Fresh ET	IVF-ET + manual acupuncture (12 acupoints, 30 min, manual stimulation q5 min) vs. sham acupuncture (4 non-acupoints, 30 min, no stimulation)	Once daily, 4 days (oocyte retrieval → ET)	ST25, ST29, ST36, SP6, LR3, CV3, CV6	Not specified	↑ VI, VFI, FI (*P* < 0.05); no diff. in endometrial thickness, CPR, LBR (*P* > 0.05)	(73)
	RCT/Ovulatory dysfunction	FET	Conventional OI ± manual acupuncture (2 acupoint sets)	Every other day, cycle Day 2 → pre-FET	BL17, BL18, BL23, BL32, GV20, GV4, SP4, SP6, KI12, GB26, CV4, CV6	Twirling; tonify CV4, CV6	↑ EIR (56.8% vs. 40.9%), ↑ CPR (66.7% vs. 40.0%), ↑ endometrial thickness (*P* < 0.05)	(74)
	RCT/PCOS	Fresh ET	Conventional GnRH-a ± manual acupuncture (two sets)	Every other day, ovulation → ET	EX-CA1, CV3, CV4, CV6, BL23, GV3, GV4, BL32	Supplement BL32, GV4, CV4, CV6	↓ E2, P; ↑ type A endometrium, CPR (72.5% vs. 48.8%) (*P* < 0.05)	(75)
	Single-blind RCT/not specified	FET	HRT ± manual acupuncture (two sets)	Once daily, menstruation → ovulation, 3 cycles	EX-CA1, ST25, ST36, SP6, SP10, LR3, CV3, CV4, CV6, CV12	Lift-thrust (ST25, CV4–CV12), twirl (ST36, SP6, LR3)	↑ LIF, integrin αvβ3, endometrial thickness & CPR (63.3% vs. 33.3%) (*P* < 0.05)	(76)
	RCT/PCOS	FET	FET ± staged manual acupuncture	3 cycles, 3 × per week	SP4, PC6, LI4, BL18, BL23, ST36, KI16, CV3, BL17, GV9	Lift-thrust, twirling	↑ CPR (67.6% vs. 38.2%), LBR (64.7% vs. 35.3%), FSH↑, E2↓ (*P* < 0.05)	(77)
Electroacupuncture (EA)	RCT/not specified	Fresh/frozen ET	IVF-ET ± EA (2 acupoint sets; 30 min)	Every other day, 3 × week, 1 month before IVF	ST40, SP6, SP10, EX-CA1, GV20, CV4, CV6, BL18, BL23	Connected to the EA apparatus post-Deqi	↓ uterine artery PI, S/D; ↑ HCG +, high-quality embryos (*P* < 0.05)	(82)
	Prospective self-controlled/DOR	Fresh ET	EA only (2 sets; 25 min)	3 cycles, 3 × per week	BL17, BL23, BL32, BL33, KI3, GV4, EX-B8, ST25, ST36, SP6, HT7, LR3	EA pairs: BL23↔BL32, ST25↔EX-CA1	↑ AMH, MII oocytes, fertilization rate (*P* < 0.05); no hormonal change	(83)
	RCT/DOR	FET	Endometrial prep ± EA (30 min)	3 cycles, 3 × per week until 1 d before ET	ST25, BL23, BL33, EX-CA1, GV4, CV4, CV6, EX-B8, HT7, SP6, KI3, LR3	EA pairs as above	↑ type A endometrium, EIR, CPR, LBR (*P* < 0.01)	(84)
	RCT/RIF	FET	HRT ± EA (20 min post-menstruation)	Every other day → ET day (1 cycle)	ST36, PC6, SP6, LR3, BL23, BL32, EX-CA1, CV4, GV20	EA to BL23 & BL32	↑ CPR (44.6% vs. 25.5%), thicker endometrium, ↓ SAS/SDS (*P* < 0.01)	(85)
TEAS	Multicenter RCT/Mixed causes (EMs, immune, ovulatory)	Fresh/Frozen ET	IVF-ET ± TEAS (before and after ET, 30 min)	24 h pre-ET & 30 min post-ET	SP8, SP10, ST29, EX-CA1, ST36, KI3, BL23, CV4, CV12	Not specified	↑ CPR (48.9% vs. 23.7%), EIR (30.8% vs. 13.9%), LBR (34.0% vs. 19.7%) (*P* < 0.05)	(90)
	RCT/not specified	Fresh ET	GnRH-a long protocol + TEAS (20–50 mA subgroups)	Daily from Day 3 → 36 h before HCG (10–13 days)	SP6, KI13, CV3, CV4, EX-CA1	Not specified	↑ Endometrial thickness (40 mA best), no effect on CPR (*P* > 0.05)	(91)
	Retrospective study/RIF	Fresh ET	TEAS (6–8 sessions per cycle + post-ET)	≥ 1 cycle before ET prep	ST25, SP6, CV3, CV4, ST36, KI3, BL23, EX-CA1	Not specified	↑ EIR, CPR, reduced biochemical pregnancy rate vs. standard IVF	(88)

RCT, randomized controlled trial; VI, vascularization index; FI, flow index; VFI, vascularization flow index; sVI, subendometrial VI; sFI, subendometrial FI; sVFI, subendometrial VFI; CPR, clinical pregnancy rate; LBR, live birth rate; EIR: embryo implantation rate; FET, frozen embryo transfer; LE, letrozole; HCG, human chorionic gonadotropin; P, progesterone; OI, ovulation induction; PCOS, polycystic ovary syndrome; E2, estradiol; E2V, estradiol valerate; LIF, leukemia inhibitory factor; PI, resistance index; RI, pulsatile index; LH, luteinizing hormone; FSH, follicle-stimulating hormone; T, testosterone; EA, electroacupuncture; b, basal; AFC, sinus follicle count; RIF, recurrent implantation failure; SAS, self-rating anxiety scale; SDS, self-rating depression scale; TEAS, transcutaneous electrical acupoint stimulation.

#### 3.1.2 Electroacupuncture

Electroacupuncture (EA) therapy activates the peripheral, segmental, and central conduction pathways by providing continuous electrical stimulation by connecting the positive and negative poles to the needle body after conventional manual acupuncture treatment (78). A clinical study (79) compared traditional manual acupuncture and EA as pretreatment for RIF patients before IVF-ET, and they found a better reduction in the proportion of type A endometrium and improved sub-endometrial blood flow in the EA pretreatment group, thus exerting a better effect on ER. In addition, the EA pretreatment group showed better vital indicators of pregnancy outcome, including EIR and CPR, compared with the traditional manual acupuncture pretreatment group. These results further support the effectiveness of EA treatment for ER in infertile women undergoing IVF-ET (Table 1).

Current clinical evidence supports the use of EA as an adjunctive treatment for infertility induced by ovulatory dysfunction. The application of EA therapy in combination with OI drugs, such as letrozole (LE) and clomiphene citrate (CC), significantly improves ovarian response, increasing ovulation rate and follicular diameter, and ultimately an increased CPR, due to a reduction in uterine arterial PI and RI, thereby improving blood flow perfusion. Moreover, E2 and P levels are improved during WOI, making EA therapy an effective adjunct to improve pregnancy outcomes by enhancing ER and promoting oocyte development and ovulation (80, 81). In a study conducted by Zhong et al. (82), 64 infertile women scheduled for IVF-ET were recruited and randomized into control and observation groups, and the observation group received additional EA pretreatment before conventional IVF-ET, lasting for one menstrual cycle from recruitment, and was administered every other day, three times a week, except during menstruation. The results showed lower endometrial PI and peak systolic velocity/diastolic velocity (S/D) in the observation group, but no statistical differences were found for endometrial thickness and morphology in intergroup comparison. Although the study failed to provide sufficient evidence to prove the efficacy of EA therapy in ER, the observation group showed a higher HCG-positive rate and a greater number of high-quality embryos, suggesting that EA therapy may improve ovarian response and oocyte quality, leading to improved pregnancy outcomes in IVF-ET. Xia's team reported that administration of EA therapy based on conventional OI treatment significantly increased AMH levels to enhance ovarian reserve function, promote oocyte development, improve oocyte quality, and ultimately increase the number of mature oocytes, fertilization, and high-quality embryos in women with diminished ovarian reserve (DOR) undergoing IVF-ET (83). Moreover, EA therapy could also improve the ER based on the conventional endometrial preparation protocol by improving the proportion of type A endometrium on ET day, thus significantly increasing the HCG positive rate, EIR, CPR, and LBR, and achieving improved FET pregnancy outcomes for DOR-induced infertility (84).

Accordingly, we conclude that EA enhances ER and improves the ovarian reserve function of DOR-induced infertility to improve pregnancy outcomes, regardless of fresh cycle ET or FET. Other advantages of EA include the improvement of endometrial thickness, as well as the reduction of self-rating anxiety scale (SAS) and self-rating depression scale (SDS) scores to alleviate anxiety in infertile women, thus achieving the goal of increasing CPR of IVF-ET (85). Furthermore, a relevant study reported that EA therapy could reduce the incidence of CC-induced side effects, including nausea, vomiting, and dermatitis, in addition to reversing its antiestrogenic effect to shorten the duration of endometrial maturation (81). These studies have expanded the indications for ER therapy, promoted its clinical application, and suggested directions for further research on mechanisms.

#### 3.1.3 Transcutaneous electrical acupoint stimulation

Transcutaneous electrical acupoint stimulation (TEAS) is a painless, non-invasive, and gentle treatment that combines transcutaneous electrical nerve stimulation (TENS) with traditional acupoints (86, 87). Self-adhesive electrodes are applied to acupuncture points instead of electrically stimulated needles, making them more acceptable to patients compared to traditional electrical stimulation treatments such as EA (88). In 2017, a group consensus (89) recognized TEAS and EA as the convergence of TCM therapy and modern technology, emphasizing their status in the field of ART. The consensus also noted that TEAS is preferred by patients due to its good operability (Table 1).

In a multicenter RCT conducted by Feng et al. (90), 739 infertile women undergoing IVF-ET were randomly assigned to either the TEAS group (*n*= 367) or the control group (*n*= 372). The TEAS group received a 30-min TEAS therapy session 24 h before and 30 min after ET. The study found that among women over 35 years old in both groups, the TEAS group had better CPR, EIR, and LBR compared to the control group. This improvement may be related to the amelioration of ER, as suggested by subsequent research (later reviewed). Another RCT conducted by Zhai et al. (91) selected 200 infertile women undergoing IVF-ET and were divided into a control group (*n*= 40) and experimental groups (divided into 4 sub-groups according to different electric stimulation intensities, with 40 patients in each sub-group). Both groups received GnRH-a long protocol as the conventional treatment and different TEAS modes: the control group received 5 mA, which had no effect by default, while the experimental groups received 20, 30, 40, and 50 mA, respectively. TEAS treatment was administered once a day for 30 min from the 3rd day of menstruation until 36 h before ovum retrieval (HCG injection day), lasting for 10–13 days. The results indicated a significant improvement in endometrial thickness in the experimental groups on the HCG injection day, confirming the efficacy of TEAS in improving ER in clinical practice. Importantly, the study found that TEAS treatment with 40 mA electric stimulation could improve ovarian conditions and result in the retrieval of more eggs, which was consistent with the results of Shuai's clinical research on TEAS improving premature ovarian insufficiency (POI) in patients undergoing IVF (92). Based on the two studies, it can be concluded that TEAS therapy improves basic endocrine indexes, enhancing ovarian reserve function through the hypothalamic–pituitary–ovarian axis (HPOA), thus improving pregnancy outcomes for IVF-ET.

Women's fertility declines with age. Poor ER conditions and follicular growth disorders are key factors in female infertility as women get older (93, 94). TEAS can improve the success rate of IVF-ET and pregnancy outcomes by improving ER and ovarian function, making it an adequate option for older women. Furthermore, relevant research has also shown the basic research value of TEAS to increase CPR and LBR in women with RIF (86, 88).

### 3.2 Clinical research of moxibustion

Moxibustion therapy has several forms, including wheat moxibustion, indirect moxibustion, and heat-sensitive moxibustion, which are commonly used in clinical practice. It provides warm stimulation to the human body, exerting its therapeutic effect of warming the channels, dissipating cold, and promoting the movement of qi and blood (Table 2).

**Table 2:** Summary of the clinical research of moxibustion.

**Type of moxibustion**	**Study design/disease**	**ART type**	**Intervention description**	**Treatment course**	**Main acupoints**	**Key findings**	**References**
Wheat moxibustion	Retrospective study/RIF	FET	Control: hormone replacement therapy (HRT) for endometrial preparation. WM group: WM added to HRT (5 cones, ~4 mg moxa each) applied on alternating acupoint sets.	Before ET: once every 2 days during the luteal phase (excluding menstruation). After ET: daily for 3 days, then every 2 days; continued until 4–5 weeks (biochemical pregnancy) or 12 weeks (clinical pregnancy).	ST36 (bilateral), CV6, CV4; ST36, EX-CA1 (bilateral).	↑ Endometrial thickness on transformation day; fewer endometrial prep cycles; ↑ biochemical pregnancy (53.3% vs. 26.7%, *P* < 0.05). No significant difference in CPR.	(95)
Herbal cake moxibustion	RCT/not specified	Fresh ET	Control: GnRH-a long protocol. Treatment: moxibustion on herbal cake (3 cones, ~2 g moxa each) added to control treatment.	Three sessions per week for 3 months (starting after menstruation), plus one session each on HCG injection day and ET day.	ST36, SP6, BL23, BL32, KI3, EX-CA1; GV14, CV4.	↑ CPR, ↑ high-quality oocytes (*P* < 0.05); ↑ endometrial thickness (*P* < 0.01), and morphology (*P* < 0.05). No difference in E2 level or biochemical pregnancy.	(96)

Wheat moxibustion, that is, applying moxibustion after rubbing a wheat-sized moxa. In a retrospective study conducted by Xiao et al. (95), 60 women with RIF who received FET were randomly selected and divided into control and wheat moxibustion groups, and the wheat moxibustion group received wheat moxibustion therapy as adjuvant treatment based on the conventional hormone replacement cycle protocol for the endometrium preparation The application of wheat moxibustion therapy before ET started from the luteal phase, once every other day, except the menstrual period; once a day for the first 3 days after ET, then once every other day. Compared to the control group, wheat moxibustion significantly reduced endometrial preparation time and increased endometrial thickness on the endometrial transformation day during the hormone replacement cycle, which improved the ER and ultimately enhanced the IVF-ET pregnancy outcomes.

Indirect moxibustion therapy is the application of moxibustion to acupoints after the placement of Chinese herbal medicine (CHM) between the moxa cones and the skin. This therapy can be named according to the type and shape of the CHM. Li et al. (96) conducted a study that assigned 66 infertile women receiving IVF-ET to the control and treatment groups, and the treatment group received moxibustion on herbal cake therapy as an adjuvant treatment based on the conventional GnRH-a long protocol. The treatment was administered in the month that received conventional treatment and 2 months before, starting from the first day after the menstrual cycle. Three discontinuous treatments were given per week until the first day of the next menstrual cycle; additionally, given once on the HCG injection day and the ET day. Compared to the control group, the treatment group exhibited superior endometrial thickness and types, as well as a higher number of high-quality ova, leading to an increase in CPR and ultimately improved IVF-ET pregnancy outcomes.

Overall, moxibustion therapy can improve ER-related parameters, thus increasing CPR in patients receiving IVF-ET and ultimately improving pregnancy outcomes. Various forms of moxibustion therapy are effective, including warm uterus moxibustion therapy, which refers to pulverizing a formula that regulates the thoroughfare and conception vessels, warms and supplements kidney qi, and regulates qi and nourishes the blood, to fill the navel, and then applying moxibustion on them. Huang et al. (97) found that warm uterus moxibustion therapy could ameliorate the ER of mild premature ovarian failure (POF) patients and improve ovarian reserve function to enhance egg quality, consistent with those of Li et al.'s research (96). Another study proposed the effectiveness of herb-partitioned moxibustion on the navel for patients with RIF receiving IVF-ET, further enriching the forms of moxibustion application to improve ER (98). Heat-sensitive moxibustion therapy may be used to improve ER (99); however, there is limited research that confirms its effectiveness in the improvement of ER, and hence further study is needed to focus on filling this gap.

### 3.3 Combination of acupuncture and moxibustion

As previously discussed, current research does not focus on using moxibustion therapy alone to improve the ER of infertile women, and it is usually combined with various forms of acupuncture treatment to enhance its therapeutic effect (Table 3). The combined therapy can better improve the ER than acupuncture, moxibustion, and Western medicine alone, and also alleviate patients' anxiety (79, 100, 101). TCM holds the belief that acupuncture can regulate the movement of qi and blood by stimulating channel qi, which can be accelerated by warm stimulation of moxibustion. Therefore, most combined therapies are used to promote endometrial blood flow status. The network meta-analysis investigated the effect of acupuncture and moxibustion therapy on intrauterine blood perfusion in patients receiving FET, and the combination of acupuncture and moxibustion therapy showed the highest efficacy in increasing the type A endometrial blood flow rate, which indicates sufficient blood supply and clear flow signals in the endometrium and sub-endometrium. Additionally, the PI and RI of uterine artery blood flow were improved, which corresponds to TCM theory (102).

**Table 3:** Summary of the clinical research of combinations.

**Combining therapy type**	**Study design/disease**	**ART type**	**Intervention description**	**Treatment course**	**Main acupoints**	**Technique/manipulation**	**Key findings**	**References**
Manually staged acupuncture + warm needling + EA.	RCT/RIF	FET	Control: hormone replacement protocol. Observation: combining therapy (manual acupuncture + warm needling + EA) added to control.	Every other day, 3 × /week, 3 cycles (one cycle = one course).	SP4, PC6; LI4, SP6, BL18 (menstrual); KI16, EX-CA1 (follicular); ST28, ST29, EX-CA1 (ovulatory); SP6, BL23, GV9 (luteal); warm needling: ST36, BL23, KI16, GV9.	Lift-thrust and twirling; slow insertion/extraction for ST28, ST29, KI16, CV3, CV12, EX-CA1.	↑ Endometrial thickness; ↑ type A/B endometria; ↑ CPR (77.8% vs. 44.4%) and LBR (75.0% vs. 36.1%) (*P* < 0.05); ↓ HAMA score & cycle cancellation (*P* < 0.05).	(101)
Traditional manual acupuncture + moxibustion	RCT/RIF	FET	Control: HRT (E2V 2 mg/d). Observation: manual acupuncture (2 alternating acupoint sets, 30 min post-Deqi) + moxibustion (30 min).	Every other day from day 5 of menstruation → 1 day pre-ET; continued 2–14 days post-ET, 3 consecutive cycles.	BL15–BL32, GV14–GV20, ST25, ST36, SP6, SP10, GV29, CV4–CV12, EX-CA1, Luanchao, Shengzhi origin; moxibustion: KI1.	Neutral supplementation & draining.	↑ Endometrial thickness (*P* < 0.01), ↑ type A morphology (*P* < 0.05), ↓ PI (*P* < 0.01), ↓ and RI (*P* < 0.05). No CPR diff. ADRs in 6 control cases, none in treatment.	(103)
Warm needling + XianYiMuCao JiaoNang	RCT/ECF	FET	Control: XianYiMuCao JiaoNang (0.8 g, 3 × /d, 3 courses). Observation: + warm needling (20 mm moxa cone on needle handle, 60 min post-Deqi).	Once daily, 3 courses (5 sessions/course, 2-day intervals).	ST29, ST36, SP6, CV3, CV4, CV6, CV12, EX-CA1.	Lift-thrust (ST29–CV12) & twirling (ST36, SP6) to neutral tonification/draining.	↓ ECF after each course; ↑ CPR (42.9% vs. 26.7%); shorter IVF-ET prep time (*P* < 0.05).	(104)
Warm needling + antibiotics & dydrogesterone	RCT/Endometritis	FET	Control: levofloxacin 0.4 g IV (3–5 d) + oral dydrogesterone 10 mg bid (14 d). Observation: + warm needling post-menstruation → ovulation (40–60 min, 20 mm moxa cone).	Both groups: 3 consecutive cycles.	ST25, ST34, ST36–ST39, CV3, CV4, CV12, EX-CA1.	Not specified.	↑ Endometrial morphology & sub-endometrial blood flow; ↑ EIR (24.4% vs. 11.9%), ↑ CPR (46.3% vs. 20.7%), ↓ early abortion (*P* < 0.01).	(106)
Warm needling + NuanGong YunZi JiaoNang	RCT/Implantation failure	FET	Control: NuanGong YunZi JiaoNang (0.45 g/pill, 3 pills tid). Observation: + warm needling (2 cm moxa cone, 50 min post-Deqi).	Once daily, 1-day interval after 4 sessions, 10 × per cycle, 3 cycles total.	ST29, GV3–GV4, CV2–CV6, BL23, BL32, KI12, KI15, EX-CA1.	Lift-thrust (ST29–EX-CA1), twirling (GV3, GV4, BL23, BL32), neutral tonification/draining.	↑ Endometrial thickness; ↑ type A morphology & blood flow; ↑ uterine perfusion; ↑ ER during WOI; ↑ CPR (47.2% vs. 33.3%) (*P* < 0.05).	(105)

HAMA, Hamilton anxiety scale; ADR, adverse drug reactions; ECF, endometrial cavity fluid.

Xue et al. (103) conducted an RCT to divide 74 patients with thin endometrium who were undergoing FET into observation and control groups. The observation group received “Tongyuan acupuncture” treatment for three consecutive months, which consisted of scientific, staged, and patterned acupuncture combined with moxibustion treatment based on the conventional hormone replacement cycle protocol. The treatment started on the 5th day of menstruation, once every other day, until the day before the ET day, and continuing the combination treatment after the ET, once every 2 days, for 14 days or until the onset of menstruation. Both groups showed increased endometrial thickness and the ratio of type A endometrium 1 d before the ET, and decreased PI and RI of the uterine spiral artery compared before treatment, though superior improvement of these parameters and higher CPR were observed in the observation group. In addition, six patients in the control group experienced adverse drug reactions (ADRs) such as breast tenderness, abdominal pain, dizziness, and nausea, while none in the observation group did, suggesting that staged acupuncture combined with moxibustion therapy could reduce the incidence of ADRs induced by Western medicine.

Warm needling is a frequently used therapy in clinical practice. It involves igniting the moxa wrapped around the needle handle after being rubbed into a ball shape or inserting a moxa stick into the needle handle to transmit heat into acupoints, thereby exerting a therapeutic effect. Studies have shown that compared with traditional acupuncture and EA therapies, this therapy has a therapeutic effect on improving ER in RIF patients by increasing the local temperature of the lower abdomen, accelerating blood circulation around the uterus and appendages, promoting endometrial fluid absorption, improving the intrauterine environment, and ultimately reducing early abortion rates. The success rate of IVF-ET can be increased by applying warm needling therapy, which can improve pregnancy outcomes (79, 104–106). In an RCT conducted by Ma et al. (107), 60 women with RIF who received FET were assigned to treatment and control groups. The treatment group received warm needling treatment based on the conventional hormone replacement protocol, starting from the second day of menstruation, once a day, until achieving clinical pregnancy, and the acupoints were adjusted after the ET. The study findings indicated that the treatment group had higher CPR and EIR, due to significant improvements in endometrial thickness and morphology, and lower RI and PI of the uterine spiral artery on the day of ET.

## 4 Mechanism research of acupuncture and moxibustion

### 4.1 Autophagy

Autophagy is a physiological cellular process that is regulated by adenosine 5′-monophosphate-activated protein kinase (AMPK) and the mammalian target of rapamycin (mTOR). Cells achieve intracellular self-regulation and substance recycling by degrading and eliminating misfolded proteins and damaged organelles. This process is crucial for adapting to starvation, development, cell death, and tumor suppression (108–110). Endometrial cells also undergo this process, which fluctuates periodically with menstruation and acts as a key factor in apoptosis (111).

Research has shown that maintaining a moderate level of autophagy is associated with successful embryo implantation and helps preserve the homeostasis of the intrauterine environment. Su et al. (112) reported a significant downregulation of autophagy-related markers after the embryo was attached to the receptive endometrium. Additionally, they observed dysregulation of decidual marker homeobox A10 (HOXA10) and progesterone receptor (PR), as well as abnormal decidualization, after administering an autophagy inhibitor. Conversely, abnormal autophagy may impair the function and activity of endometrial cells, thereby affecting embryo implantation (113). Yang et al. (114) conducted a study in which they observed more autophagosomes in the mid-secretory endometrium of RIF patients, which was accompanied by downregulation of the activation of the autophagy-related signaling pathway and decreased expression of ER-related parameters. Zhu et al. (115) found differential gene expression in RIF patients, including increased autophagy markers such as microtubule-associated protein 1 light chain 3II (LC3-II), lysosomal-associated membrane protein 2 (LAMP2), and hypoxia-inducible factor-1α (HIF-1α), as well as drastically downregulated P62. Similar results were observed at the protein level, and the autophagy puncta were markedly enhanced in the endometrial tissue of RIF patients.

Based on these studies, it appears that autophagy activity is closely correlated with endometrial receptivity, and maintaining a balanced autophagic level may support optimal endometrial function. However, the evidence remains largely correlative, and a direct causal link between autophagy modulation and improved implantation outcomes has not yet been firmly established.

Qi et al. (116) conducted a study where they randomly divided 40 thin endometrium patients into treatment and control groups, each with 20 cases, and selected 20 patients with normal endometrium during the same period as the normal group. All three groups received FET as the conventional treatment, with the treatment group also receiving intracavitary physiotherapy combined with acupuncture treatment, which involved the use of an intracavitary physiotherapy machine that transmitted vibration waves of different frequencies through two electrodes placed on both sides of the lower abdomen. These waves were delivered to a heated physiotherapy probe inserted into the vagina (after disinfection) to produce a localized vibration and massage effect. The therapy focuses on the uterus as the core and uses acupuncture to provide strong stimulation, effectively promoting endometrium regeneration. The treatment began on the first day of the menstrual cycle and continued every other day for 30 min until the simulated transplantation day. During this time, a small amount of endometrial tissue was extracted for follow-up research. The study found that the combined therapy significantly increased endometrial thickness and the ratio of type A endometrial cells in patients with thin endometrium while decreasing bilateral uterine artery PI, RI, and S/D. Additionally, the therapy was associated with upregulated mRNA and protein expression levels of HOXA10 and mTOR and downregulated AMPK in endometrial tissue, as well as improved endometrial and clinical pregnancy rates (EIR and CPR) compared to the control group. Importantly, no statistically significant differences were observed when compared to the normal group, indicating that the observed improvements may be linked to modulation of the AMPK/mTOR pathway and autophagy-related markers. However, whether this reflects a direct causal mechanism or a secondary physiological response remains to be confirmed by further mechanistic and clinical studies. Thus, acupuncture combined with intracavitary physiotherapy may contribute to improved endometrial receptivity through multiple pathways, but the exact molecular mechanisms require additional verification.

### 4.2 Pinopodes and their related molecules

A connection is established between the trophectoderm of blastocysts and the endometrial luminal epithelium during embryo implantation. Therefore, changes in endometrial epithelial cell-related molecules greatly impact the ER (117, 118). For example, pinopodes—cellular protrusions of the apical plasma membrane of uterine epithelial cells—are typically expressed during the middle to late secretory phases and are considered morphological indicators of the window of implantation (WOI) (119, 120). Pinopodes development is regulated by estrogen and P, particularly the latter. An upregulated level of progesterone and a downregulated progesterone receptor B appear to promote their formation (121–123). Additionally, pinopodes undergo menstruation-dependent morphological changes (120). Although the appearance of pinopodes is frequently correlated with enhanced endometrial receptivity, their role as a definitive predictor of implantation success remains debated. Current evidence suggests an associative rather than causal relationship between pinopode formation and embryo implantation. Further studies are required to clarify whether pinopodes actively facilitate embryo adhesion or simply reflect a receptive endometrial state.

Integrins and related molecules are considered potential biomarkers for evaluating and predicting ER and WOI (124). These biomarkers are closely associated with molecules such as integrins, LIF, and HOXA10. Integrins, which belong to the adhesion molecule family, are composed of α and β subunits that have various combinations and functions (125). In addition, integrins are primarily found in endometrial, decidual, and extravillous cytotrophoblast cells, where they are involved in intercellular and extracellular adhesion and influence changes in the endometrial phenotype during the secretory phase, the first stage of implantation (126). The integrin alphavbeta3 (αvβ3), which consists of αv and β3 subunits, is considered a potential receptor for blastocyst attachment and is expressed in the WOI (127, 128). Immunostaining results indicate that αvβ3 expression is periodic, as evidenced by the increase in αv subunits throughout the menstrual cycle and the sudden appearance of β3 subunits on luminal and glandular epithelial cells on day 20 of the menstrual cycle (129). Moreover, studies have shown that the β3 subunit is expressed in most glandular epithelial cells of women with normal fertility but is downregulated in the endometria of infertile women during the WOI and has delayed immunostaining in the luteal phase (129, 130). Thus, some researchers have proposed β3 as a potential biomarker to evaluate ER and determine the optimal time for ET in ART (131). Regarding the expression level of αvβ3, in addition to the upregulation stimulated by heparin-binding epidermal growth factor (HB-EGF) and epidermal growth factor (EGF) and the downregulation affected by E2 (128, 132), it is also related to the downregulated P receptor (133). Studies have shown that αvβ3 and its ligand, osteopontin (OPN), which is directly regulated by P stimulation, may co-localize in the endometrium and exert effects on ET (128, 132). In addition, αvβ3 has a high predictive value for the ER (15, 132), depends on the adhesion of early-stage pinopodes to exert its effect, and is localized to mid-luteal pinopodes (134). In particular, disruption or downregulation of αvβ3 function has been associated with poor ER and the incidence of infertility (129, 135). Although αvβ3 expression has been consistently correlated with the receptive phase of the endometrium and is widely regarded as an important biomarker, direct causal evidence demonstrating that its modulation leads to successful implantation remains limited. Current data primarily support an associative relationship between integrin expression and endometrial receptivity, and further mechanistic and clinical studies are required to confirm whether these molecular changes actively contribute to implantation or merely reflect a receptive endometrial state.

LIF is a pleiotropic cytokine of the LI-6 family that can be secreted by pinopodes and is expressed in a menstruation-dependent manner, gradually increasing in the mid to late secretory phase and peaking during implantation (136–138). As a key regulatory factor for endometrial function, LIF plays an important role in implantation and appears to regulate human embryo implantation in a paracrine or autocrine manner (139–142). Quantitative real-time PCR (qPCR) confirmed that LIF was involved in the establishment of the ER, based on the changes in gene expression levels before and after the process (143). Furthermore, the protein concentration of LIF during WOI is confirmed to predict the ER of IVF with fresh blastocyst transfer (144). The combination of LIF and the receptor complex gp130 and LIFRβ on the cell surface forms a trimeric signal transduction complex, then undergoes phosphorylation after combining with Janus kinases 1 (JAK1), recruiting STAT family members such as STAT3 to form the LIF-JAK1-STAT3 signaling pathway, thus initiating the signal cascade response (142, 145, 146). It has been reported that reduced activation of this signaling pathway is associated with recurrent implantation failure (RIF) (147). In addition, LIF is also considered a biomarker for infertility associated with EMs (148, 149). Although LIF expression and signaling have been consistently correlated with endometrial receptivity and implantation outcomes, current evidence primarily supports an associative relationship. Further mechanistic studies are needed to determine whether alterations in LIF signaling play a causal role in implantation success or serve as indicators of a receptive endometrial state.

The human endometrium has a central HOX signaling pathway to regulate the establishment of the ER (150). For example, HOXA10, a biomarker associated with embryo implantation, is periodically expressed in the human endometrium and appears to participate in the implantation process by influencing downstream gene expression. Its expression level peaks during the WOI, likely in response to the regulatory effects of estrogen (E2) and progesterone (P) on ER establishment (151–153). The putative ameliorative effect of HOXA10 on the ER can be divided into two aspects: promoting the development of pinopodes and increasing their expression level (154, 155); upregulating the expression of E-cadherin, which belongs to the family of cellular adhesion molecules (156). Furthermore, HOXA10 has been proposed as a potential marker for endometriosis (EMs), as its reduced expression is associated with the occurrence of this condition (151, 153, 157, 158).

Animal experiments have shown that both traditional manual acupuncture and electroacupuncture may enhance endometrial receptivity (ER) by modulating the number of pinopodes and the expression levels of related molecules, which have been associated with increased embryo implantation and pregnancy rates in experimental models (159, 160). Hu et al. (122) conducted a study on controlled ovarian hyperstimulation (COH) rats and found that traditional manual acupuncture could restore E2/P balance, appeared to correct the abnormal development of pinopodes, and was associated with elevated expression levels of pinopode-related molecules, including LIF and integrin-β3. These changes were accompanied by improvements in ER and a forward shift of the WOI, ultimately resulting in an increased pregnancy rate. After applying high-frequency electroacupuncture treatment to the COH rats, You et al. (161) found that the treatment increased the number of pinopodes and the expression of the LIF/STAT3 signaling pathway. Additionally, it was found to reduce the protein expression levels of E-cadherin, β-catenin, and CLDN1, thereby potentially facilitating epithelial cell dissociation and enhancing the conditions favorable for blastocyst implantation. These effects were associated with improved ER and a higher number of implantation sites. While these animal studies provide valuable insights into potential mechanisms, the observed effects should be interpreted as associative rather than definitively causal. Further mechanistic validation and clinical studies are required to determine whether the modulation of pinopode-related signaling pathways by acupuncture directly contributes to improved endometrial receptivity in humans.

The results of these animal experiments have also been validated in clinic-based studies. In a study conducted by Wang et al. (76), ER-related factors were measured in both groups before and after the interventions. It was found that compared to the control group that received only oral Western medicine, the treatment group that received traditional manual acupuncture in addition to oral medication showed significantly higher expression levels of LIF and integrin αvβ3. Feng et al. (90) recruited 120 women with FET to conduct mechanism research and randomly divided them into control and TEAS groups. The TEAS group received TEAS treatment on the 16th and 17th day of the menstrual cycle, as previously described. The study reported that the TEAS group exhibited better-developed pinopodes and was associated with significantly increased levels of integrin α1β/αvβ3, LIF, and progesterone (P) compared to the control group. These findings provide preliminary clinical evidence consistent with the molecular changes observed in animal studies; however, they primarily demonstrate associative relationships rather than direct causation. Further large-scale, well-controlled mechanistic studies are needed to clarify whether acupuncture and TEAS directly modulate endometrial molecular pathways or indirectly influence them through systemic endocrine and vascular regulation.

### 4.3 Non-coding RNA

In eukaryotes, only about 2% of genes can be transcribed and translated into proteins. The majority of gene transcripts do not encode proteins and are referred to as non-coding RNA (ncRNA). Studies have shown that ncRNAs participate in various biological processes, including the regulation of gene expression at the transcriptional, RNA processing, and translational levels, as well as contributing to genome protection against foreign nucleic acid invasion (162). Despite significant progress in understanding their roles, the precise molecular functions and regulatory networks of ncRNAs in endometrial receptivity remain incompletely understood, and several challenges continue to exist (163, 164).

Most current research has focused on exploring microRNAs (miRNAs) that may participate in regulating the establishment of endometrial receptivity (ER). Over 20 related miRNAs have been discovered in human studies. For example, miR-30d-5p, which belongs to the miR-30 family, has been proposed as a potential biomarker of ER (165, 166). MiRNAs are found in endometrial epithelial cells, stromal cells, and other locations, such as uterine fluid (UF), that are involved in the microenvironment of the uterine cavity. They have been extensively profiled in UF during the receptive phase (166–168). MiRNAs are believed to act as regulatory molecules during the complex process of embryo implantation (169). A recent study discovered that miRNAs were secreted by human blastocysts, taken up by endometrial epithelial cells, and induced changes in endometrial functions (170). These changes included potential interactions with HOXA10 and steroid hormones, which may influence the development of pinopodes and the secretion of LIF. Additionally, miRNAs were found to be involved in the establishment of ER, embryo implantation, and embryo-endometrium interaction (171–173). Differentially abundant miRNAs have been found in RIF women (165, 168). Certain endometrial miRNAs have been suggested as non-invasive biomarkers for diagnosing and managing RIF, as well as predicting IVF-ET success rates (174). Moreover, miRNAs may serve as biomarkers for the early diagnosis of endometriosis (EMs) (175). For instance, miR-543 has been confirmed to have a low expression level during the WOI in EMs-related infertile women, which could affect the ER (176). Mu et al. (177) analyzed miRNA samples from infertile women who received or did not receive acupuncture treatment. They found that has-miR-449a, has-miR-3135b, and has-miR-345-3p may be potential targets to increase and promote the ER of patients undergoing IVF-ET. These miRNAs were implicated in pathways such as endocytosis, axon guidance, oxytocin signaling, the Hippo pathway, and estrogen signaling. Furthermore, a study by You et al. (178) showed that EA therapy, especially high frequency (continuous wave, 50 Hz, 1 mA), could suppress miR-223-3p expression in the endometrium of COH rats. This modulation appeared to enhance the LIF/STAT3 signaling pathway and was correlated with improved ER. In addition, EA therapy was observed to reduce the expression of cell adhesion molecules, including E-cadherin, which may facilitate blastocyst implantation by lowering mechanical barriers and increasing the number of endometrial pinopodes.

lncRNAs are potential biomarkers for ER due to their association with successful embryo implantation. However, current studies remain largely at the omics stage, mainly focusing on screening differentially expressed genes using bioinformatics approaches. The relationship between lncRNA and poor ER remains unclear (167, 179, 180). Feng et al. (181) found that their screened differentially expressed genes associated with RIF and recurrent miscarriage (RM), which were predicted to be involved in uterine immune regulation, growth factor binding, vascular proliferation, apoptosis, and steroid biosynthesis—processes potentially contributing to endometrial preparation for embryo implantation. Cai et al. (182) conducted a study suggesting that altered expression of screened lncRNAs may be involved in EMs-related inflammation and could affect ER during the WOI in EMs rats, possibly contributing to implantation failure. Wang et al. (183) first discovered the expression of lncRNA TCL1 upstream neural differentiation-associated RNA (TUNAR) in human endometrium. Their findings indicated that lncRNA TUNAR might play a role in embryo implantation by potentially regulating blastocyst attachment to the endometrial epithelium and influencing the proliferation and decidualization of endometrial stromal cells (ESCs). Furthermore, a recent study reported that upregulation of HOXA10 promoter activity and expression through lncRNA nuclear paraspeckle assembly transcript 1 (NEAT1), in conjunction with CCCTC-binding factor (CTCF), was associated with enhanced proliferation of endometrial epithelial cells (EECs) and improved establishment of ER, which may facilitate embryo implantation (184). However, we were unable to find any high-quality research on the impact of acupuncture and moxibustion therapy in ameliorating ER, specifically targeted by lncRNA, despite retrieving related literature from the past 5 years. The abovementioned findings highlight the potential of lncRNA as a mechanistic target and underscore the need for future research exploring specific molecular pathways and acupuncture-related effects.

CircRNA is a newly discovered loop-structured ncRNA with significant research potential. Although only a few studies have been conducted to date, emerging evidence suggests that circRNAs may be involved in female reproductive-related diseases such as endometriosis (EMs) (185). Zhao et al. (186) reported that hsa_circ_001946 might influence endometrial receptivity (ER) by interacting with miR-135b and increasing HOXA10 expression, thereby suggesting a possible regulatory role of circRNAs in molecular signaling networks. Furthermore, Shen et al. (187) conducted high-throughput RNA sequencing and bioinformatics analysis on samples from six pairs of patients who were either treated with acupuncture and moxibustion or not. The results indicated that acupuncture and moxibustion therapy may modulate ER-related molecular pathways through alterations in circRNA expression, and suggested potential interactions within the circRNA–miRNA–mRNA regulatory network. Future studies should aim to further elucidate the relationship between ER and circRNAs, with particular focus on the possible mechanisms through which acupuncture might influence ER via circRNA-mediated signaling pathways. Overall, current findings on circRNAs in endometrial receptivity are preliminary and primarily associative. Although early studies imply that acupuncture and moxibustion could modulate circRNA-related molecular networks, the causal mechanisms remain hypothetical and require validation through rigorous mechanistic and clinical investigations.

### 4.4 VEGF

VEGF is a multi-functional factor that primarily regulates the proliferation, differentiation, and survival of endothelial cells and vascular permeability (188). The VEGF family consists of several growth factors, including VEGF-A, VEGF-B, VEGF-C, and placental growth factor (PLGF). Among these, VEGF-A—commonly referred to simply as VEGF—plays a predominant role in promoting vasculogenesis and angiogenesis and in modulating vascular permeability (189).

The interaction between VEGF and its receptor VEGFR-2 is known to activate the phosphatidylinositol-3-kinase (PI3K)–AKT signaling pathway, thereby promoting the production of endothelial nitric oxide synthase (eNOS) and the formation of nitric oxide (NO) (190–192). This cascade contributes to a range of physiological processes, including the proliferation and differentiation of endothelial cells, tube formation, angiogenesis, and regulation of vascular permeability (193). Moreover, VEGF has been shown to play a key role in endometrial angiogenesis during the menstrual cycle and in the “guidance” of vascular growth during embryo implantation (194). Its expression is regulated by both steroid hormones and the local factors that are independent of it, in a menstrual cycle-dependent manner. Specifically, VEGF expression appears to depend on the modulation of estrogen (E2) and/or progesterone (P), particularly during the later stages of the menstrual cycle (195).

Consequently, VEGF is considered a crucial biomarker that reflects or correlates with the endometrial receptivity (ER) condition (196), and its positive association with ER has been reported in several studies (197). It has been reported that VEGF promotes angiogenesis to ensure adequate blood supply to the endometrium. Conversely, a deficiency in angiogenesis-related markers closely associated with VEGF has been suggested to contribute to thin endometrium–related infertility (198). For instance, LIF treatment can enhance the VEGF production of the EMs representative cell lines (199), and the integrins αvβ3 can combine with the VEGFR-2 to form a co-receptor (200). In addition, certain miRNAs such as miR-16-5p have been implicated in the regulation of angiogenesis (201), and inhibition of miR-494-3p has been associated with upregulation of VEGF and LIF protein levels, as well as an increased p-mTOR/mTOR ratio in endometrial tissue (202).

Traditional manual acupuncture has been reported to be associated with increased VEGF expression levels (122). Xing et al. (159) observed that traditional manual acupuncture might ameliorate angiogenesis disorders and modulate the expression of pinopode-related markers through activation of the PI3K/AKT signaling pathway. This regulation was associated with elevated gene and protein expression levels of VEGF, VEGFR2, PI3K, AKT, and phosphorylated AKT (P-AKT), as well as increased eNOS and nitric oxide (NO) levels in the endometrium of rats with polycystic ovary syndrome (PCOS). These molecular changes coincided with improvements in endometrial receptivity (ER) and an increased number of implantation sites. Furthermore, in a comparative study, Hu et al. (203) found that wheat moxibustion therapy—when compared with conventional estrogen-based therapy—was associated with greater endometrial thickness and upregulated expression of VEGF, keratin, and vimentin in the endometrium. The treatment also correlated with increased expression of ER-related factors such as HOXA10 and LIF, suggesting a potential role in endometrial repair and enhancement of ER. Together, these findings suggest that acupuncture and moxibustion may influence endometrial angiogenesis and receptivity through modulation of the VEGF–PI3K/AKT–eNOS signaling pathway. However, these observations are primarily derived from preclinical and small-scale studies, and the causal mechanisms by which acupuncture or moxibustion affect VEGF-mediated molecular pathways remain to be conclusively demonstrated in clinical and mechanistic research.

## 5 Discussion

Acupuncture and moxibustion therapy regulate the ER-related factors, encompassing the endometrial thickness, blood flow perfusion, volume, and types, to increase the number of embryo implantations, CPR, and LBR, improving the IVF-ET outcomes in the end. The underlying mechanisms are closely associated with endometrial autophagy regulation, as well as the expression levels of pinopodes and their related molecules, ncRNA, and angiogenesis-related molecules. This literature review compiles relevant studies from the past 5 years on the use of acupuncture and moxibustion therapy to improve the ER of infertile women receiving IVF-ET, and found controversies that we have divided into two aspects: safety and efficacy evaluation of the acupuncture and moxibustion therapy, and the identification of definite ER-related factors.

To evaluate the safety and efficacy of acupuncture and moxibustion therapy as an adjuvant treatment in the ER of infertile women with IVF-ET, we screened systematic reviews (SRs) and meta-analyses (MAs) on the relevant topic published within the last 5 years. Studies have shown that acupuncture and moxibustion therapy can improve IVF-ET pregnancy outcomes by enhancing CPR (52, 204–207), EIR (204, 205, 207), LBR (205, 206), and ongoing pregnancy rate (205) which may be associated with better improved ER-related parameters, encompassing the endometrial thickness (52, 204, 207), morphology (52, 207), and blood flow condition (207). Nonetheless, some of the literature supporting this argument has low quality of included RCTs, and controversies exist between different research (52, 204, 207). Additionally, a study included an RCT treated with flash cupping in combination with acupuncture and moxibustion in the acupuncture group, while the placebo group did not receive adjuvant treatment, which failed to confirm the efficacy of acupuncture and moxibustion therapy and may have reduced the confidence of the results, although other included RCTs were high-quality (43). Moreover, we noticed that the research conducted by Coyle et al. (208) included 8 RCTs with mild to moderate to high certainty evidence and concluded that acupuncture treatment did not demonstrate any statistically significant effect on IVF outcomes compared to placebo acupuncture. Additionally, moderate adverse events associated with acupuncture, such as discomfort and bruising, were reported. Quan et al. (205) noted that the acupuncture group had slightly more adverse events, such as local pain, bleeding, bruising, and pruritus, compared to the control group, but the differences between studies were moderate. Hu et al. (209) reevaluated current SRs and MAs and found that most of them provided low-quality evidence. Furthermore, present studies lack SRs and MAs focused on specific types of acupuncture; we only found two SRs/MAs (86, 87) of TEAS, and there were no SRs and MAs focused on the evaluation of moxibustion therapy. Accordingly, more high-quality studies are needed to thoroughly evaluate the efficacy and safety of these treatments.

Apart from its direct physiological effects on endometrial blood flow and hormone regulation, acupuncture may also exert indirect benefits by reducing psychological stress and anxiety levels in women undergoing IVF-ET. Several studies have demonstrated that acupuncture can modulate the hypothalamic–pituitary–adrenal (HPA) axis, decrease cortisol secretion, and enhance autonomic nervous system balance, thereby alleviating anxiety and tension associated with assisted reproduction. Moreover, improved psychological wellbeing may positively influence the uterine environment and implantation potential. Therefore, the stress-reducing and mood-stabilizing effects of acupuncture might represent an important indirect mechanism contributing to improved endometrial receptivity and IVF-ET outcomes.

Additionally, some Chinese researchers have not adhered to the “standards for reporting interventions in clinical trials of acupuncture (STRICTA)” and have failed to conduct relevant RCTs following these guidelines, as evidenced in some literature where there is incomplete consistency between results and conclusions (73) and the absence of the information regarding the duration of acupuncture treatment (76), which may cause (false) negative results thus increasing the difficulties for subsequent researchers to replicate the study. Importantly, acupuncture and moxibustion therapy differ from modern medical therapies as they are based on the principle of “point selection based on pattern differentiation,” which mostly depends on the professional skills of acupuncturists. Therefore, it is important to note that subjective evaluations should be excluded unless marked as such, and it is possible for different research teams targeting the same indicator to obtain opposite results. For instance, Wang et al. (210), Ma et al. (107), and Luo et al. (106) conducted studies that demonstrated the effectiveness of warm needling in improving the ER; however, the changes in endometrial thickness are varied: both the control and treatment groups showed an increase, but no statistical differences when compared to the control group in Wang et al.'s study; in Ma et al.'s study, both two groups showed a statistically significant increase compared to the control group; in Luo et al.'s study, and no changes in either group after interventions. Furthermore, clinical research has shown that despite the acupuncture and moxibustion therapy can enhance the ER of infertile women receiving IVF-ET, no statistical differences in key outcome indicators such as CPR, LBR, and miscarriage rate compared to the control groups LBR, and miscarriage rate showed no statistical differences when compared with the control groups (73, 91), which is closely related with lacking large-scale, multicenter, and high-quality RCTs conducted domestically (204). Currently, little research with high quality is centered on confirming the efficacy of moxibustion therapy alone, and the most relevant studies focus on the mechanism of moxibustion therapy as a complement to enhance the efficacy of acupuncture therapy. Some researchers have conducted high-quality studies focused on exploring the effects of different types of moxibustion applications; however, lacking systematic research to discover the specific mechanisms of moxibustion therapy itself on ameliorating the ER.

Interestingly, acupuncture and moxibustion treatment demonstrated an improved biochemical pregnancy rate in our included RCT (88), which is consistent with the results of relevant SRs/MAs (52, 204, 205), suggesting that acupuncture and moxibustion therapy might not promote embryo development after biochemical pregnancy and have no significant effect on the improvement of ER (95). Nonetheless, Xing et al.'s (77) clinical study on manually staged acupuncture for FET of PCOS exhibited the opposite result, that it could decrease the biochemical pregnancy rate compared with the given FET protocol only. Accordingly, follow-up research should conduct more RCTs with multicenter and large-scale samples to determine the efficacy of staged acupuncture or find other strategies to increase the CPR, in favor of improving the acceptance of acupuncture and moxibustion therapy. Finally, future research is needed to perfect the mechanism of acupuncture and moxibustion therapy in the ER of infertile women receiving IVF-ET, such as the recently discovered circRNA that was described before. Additionally, Yuan et al.'s (202) study confirmed that miR-494-3p regulated the ER in mice via PI3K/AKT/mTOR signaling pathway, and another study conducted by them showed that the combination therapy of acupuncture and Chinese compound medicine also improved the ER by activating PI3K/Akt/mTOR signaling pathway to inhibit miR-494-3p/HOXA10 axis (211). Importantly, miR-494-3p has been confirmed to inhibit mitophagy in cardiomyocytes (212). In the next step, we can discuss whether acupuncture and/or moxibustion therapy regulates PI3K/AKT/mTOR/miR-494-3p-mediated endometrial autophagy to improve the ER.

Regarding ER-related factors, Mayer et al. (67) concluded that endometrial and sub-endometrial vascularization parameters should not be used to predict pregnancy outcomes because no differences were observed in an FET cycle, consistent with Liu et al.'s conclusion (68). In addition, they found endometrial thickness was not a reliable predictor of IVF-ET pregnancy outcomes. Zhang et al. (64) suggested that uterine and endometrial spiral artery RI were more suitable for evaluating the ER than the PI and S/D. Some opinions indicated that endometrial volume was not suitable for predicting pregnancy in single blastocyst ET cycles (213). Instead, it has been reported that the evaluation of types of endometria could be used to assess the ER and predict the IVF-ET pregnancy outcomes (214). Follow-up research should discuss and decide on a set of feasible standard plan schemes to determine the definite factors that affect the ER and even predict the IVF-ET pregnancy outcomes, which will provide a better understanding of the effects of acupuncture and moxibustion on the ER of infertile women receiving IVF-ET. Moreover, although numerous studies have shown that acupuncture and moxibustion can improve ER-related parameters such as endometrial thickness, morphology, and vascularization, these improvements do not always translate into higher clinical pregnancy or live birth rates. Some reports [e.g., Mayer et al. (67) and Liu et al. (68)] found no significant correlation between endometrial vascularization and pregnancy outcomes in FET cycles, while others [e.g., Zhang et al. (64)] indicated that specific morphological and hemodynamic features may better reflect implantation potential. Therefore, large-scale, well-designed clinical trials are still needed to clarify whether ER enhancement consistently leads to meaningful clinical benefits.

Despite the encouraging evidence, previous studies have reported inconsistent or even controversial results regarding the efficacy of acupuncture and moxibustion in improving ER. Several factors may explain these discrepancies. First, small sample sizes in many randomized controlled trials (RCTs) may have reduced statistical power, resulting in inconclusive or variable outcomes. Second, there is a lack of standardization across studies with respect to intervention protocols, including acupoint selection, stimulation intensity, treatment duration, and combination with other therapies. These differences increase heterogeneity and make direct comparison challenging. Third, variation in acupuncturist skill and clinical experience may contribute to differing therapeutic effects. The subtlety of needling depth, manipulation technique, and individual interpretation of traditional diagnostic principles can influence outcomes significantly. Fourth, methodological limitations such as inadequate randomization, insufficient blinding, and incomplete reporting increase the risk of bias. Finally, heterogeneity in outcome assessment—including the use of different imaging parameters, hormonal profiles, or molecular markers to evaluate ER—further complicates the interpretation and synthesis of results. Collectively, these factors likely account for the inconsistent findings across the literature. Therefore, future research should prioritize larger, multicenter RCTs with standardized treatment protocols, qualified acupuncturist training, and consistent outcome measures to enhance the reliability and reproducibility of evidence.

This review has several limitations that should be acknowledged. First, although the literature search was conducted systematically across multiple databases, publication bias may still exist, as studies with negative or inconclusive results are less likely to be published. Second, the methodological quality of the included studies varied considerably. Many randomized controlled trials (RCTs) lacked adequate randomization and blinding procedures, and sample sizes were generally small, which may increase the risk of selection and performance bias. Third, differences in acupuncture and moxibustion protocols—including acupoint selection, stimulation parameters, frequency, and treatment duration—made direct comparison between studies challenging. Moreover, most studies focused on short-term outcomes, and few included long-term follow-up or live birth rates, limiting the assessment of sustained effects. Finally, because this review adopted an evidence-based narrative approach rather than a full systematic meta-analysis, the synthesis was primarily qualitative, and quantitative heterogeneity could not be evaluated.

Despite these limitations, this review provides a comprehensive summary of current evidence on acupuncture and moxibustion therapy for improving endometrial receptivity in IVF-ET, and highlights the need for future high-quality, multicenter, and standardized clinical trials to validate these findings.

## 6 Conclusion

Acupuncture and moxibustion may serve as effective adjunctive therapies to enhance endometrial receptivity (ER) in IVF-ET, with consistent evidence showing improvements in ER-related parameters and pregnancy outcomes via mechanisms including autophagy modulation, pinopode regulation, and VEGF-mediated angiogenesis. Nevertheless, further high-quality, multicenter RCTs with standardized protocols are essential to confirm their efficacy, resolve existing controversies, and reduce bias in future evidence, ultimately promoting their safe and widespread clinical application.
