# Supplementary material for: Effects of acupuncture and moxibustion therapy on endometrial receptivity of infertile women with in vitro fertilization–embryo transfer: status quo and countermeasures
Source: Front Med (Lausanne). 2026 Feb 10;12:1702214. doi: 10.3389/fmed.2025.1702214 (PMC12929554; doi:10.3389/fmed.2025.1702214)
Supplement: Supplementary file 1 [file Table_1.pdf]

Supplementary Table S1: Risk of Bias Summary of Included Studies

Study Type	Number of Studies	RoB 2.0/NOS Score Distribution	Key Bias Sources
RCTs	146	Low: 32; Moderate: 101; High: 13	Incomplete blinding (n=89); attrition bias (n=37)
Non-RCTs	68	NOS ≥7: 63; NOS <7: 5	Selection bias (n=5); unadjusted confounders (n=3)
